# Two generic mechanisms for emergence of direction selectivity coexist in recurrent neural networks

**DOI:** 10.1186/1471-2202-14-S1-P204

**Published:** 2013-07-08

**Authors:** Dmitry Tsigankov, Matthias Kaschube

**Affiliations:** 1 Frankfurt Institute for Advanced Studies, Frankfurt, 60438, Germany

## 

In the mammalian visual cortex, the time-averaged response of many neurons is maximal for stimuli moving in a particular direction. Such a direction selective response is not found in LGN, upstream of the visual processing pathway, suggesting that cortical networks play a strong role in the generation of direction selectivity. Here we investigate the mechanisms for the emergence of direction selectivity in the recurrent networks of nonlinear firing rate neurons in layer 4 of V1 receiving the input from LGN. In the model the LGN inputs are characterized by different receptive field positions, and their relative temporal phase shifts are reversed for the stimuli moving in the opposite direction. We propose that two distinct mechanisms result in the neuronal direction selective response in these recurrent networks. The first one is a result of nonlinear feed-forward summation of several time-shifted inputs. The second mechanism is based on the competition between neurons for firing in a winner-take-all regime. Both mechanisms rely on inhibitory interactions in the connectivity matrix of lateral connections, but the second one involves inhibitory loops. Typically, the first mechanism results in lower selectivity values than the second, but the time-course of acquiring direction selective response is faster for the first mechanism. Importantly, the two mechanisms have different input frequency tuning. The first mechanism, based on the nonlinear summation, result in a relatively narrow tuning curve around the preferred frequency of the stimulus in the case of the moving grating. In contrast, the direction selectivity arising from the second mechanism depends only weakly on the input frequency, i.e. has a broader tuning curve. These differences allow us to provide the recipe for identifying in experiment which of the two mechanisms is used by a given direction selective neuron. We then analyze how the statistics of the connections in the random recurrent networks affect the relative contributions from these two mechanisms and determine the distributions of the direction selectivity values. We identify the motifs in the connectivity matrix, which are required for each mechanism and show that the minimal conditions for both mechanisms are met in a very broad set of random recurrent networks with sufficiently strong inhibitory connections. Thus, we propose that these mechanisms coexist in generic recurrent networks with inhibition. Our results may account for the recent experimental observations that direction selectivity is present in dark-reared mice and ferrets [1,2]. It can also explain the emergence of direction selectivity in species lacking a spatially organized direction selectivity map.
